# Sir Napier Burnett and Employers' Subscriptions

**Published:** 1920-07-17

**Authors:** 


### NEW HOSPITALS AND THEIR FINANCE.

### Sir Napier Burnett and Employers' Subscriptions.

The meeting lately held to establish a county hospital in Rutland was addressed by Sir Napier Burnett in an interesting speech which touched several matters of general policy. Colonel Gretton first explained that the lease of the building occupied at present by the cottage hospital would expire in 1924, and there was over £3,400 in hand towards a building for which Lord Lonsdale had already offered a site. It had been hoped to provide sixteen beds in the men's and women's wards, accommodation for maternity cases, and a children's block; but the cost of £8,000 was prohibitive. On the other hand, the lease of the building might be renewed and the building itself could be transformed.

Sir Napier Burnett advised his hearers not to spend all the money in stone and lime, or, indeed, upon any permanent building. A central block would allow expansion in any direction and for any purpose, as the changing needs of the time suggested. The additions could be of wood. He advocated a maternity section at once, and a separate portion for children. The conveniences of committees and of medical men, for which hospitals had once been held to exist, must come second to consideration for the patient. The State might have something to say in regard to hospitals which appeared to like to be in debt.

One would imagine, he added, that some hospitals were in liquidation, but the figures before him showed that during the war hospitals, in the mass, had had a surplus of £350,000. During the war they had been "subsidised" by grants for the treatment of the wounded, but in the past year very few showed a balance in hand. The deficit of most hospitals was due to a stationary income and an inflation of prices. He believed the system of appealing for money to be dead, and that the time had come for hospitals to be frank about their finances and for employers and their workpeople to agree to a voluntary levy. If private support was withdrawn and the State managed the hospitals they would be governed by a growing bureaucracy, and saddled with rising rates and heavier taxes.

After some discussion a Rutland Cottage Hospital Fund was formed and a committee appointed.

